# Multidimensional urban morphology and carbon intensity: Nonlinear drivers in dense cities

**DOI:** 10.1016/j.isci.2026.115762

**Published:** 2026-04-16

**Authors:** Jiangkun Zhu, Zhanxiang Chen, Gangyi Tan

**Affiliations:** 1 School of Architecture and Urban Planning, Huazhong University of Science and Technology, Wuhan 430074, China; 2 Hubei Engineering and Technology Research Center of Urbanization, Wuhan 430074, China; 3 Hubei Rural Construction Center, Wuhan 430074, China; 4 HUST Built Heritage Research Center, Wuhan 430074, China

**Keywords:** environmental science, machine learning, research methodology, social sciences

## Abstract

Urban morphology (UM) significantly influences carbon emissions (CEs), yet fine-scale mechanisms involving three-dimensional (3D) structure and natural substrates remain a “black box.” Using refined block delineation with explainable machine learning (XGBoost-SHAP) to analyze 708 functionally distinct blocks in Wuhan, a high-density metropolis. Results indicate that natural geographic elements, 3D morphology, and human activity intensity outweigh traditional two-dimensional (2D) metrics as drivers. We identified three core mechanisms: (1) Geographic constraints dominate, with slope as the primary driver whose interaction with floor area ratio (FAR) reveals synergistic risks. (2) Nonlinear thresholds exist, exemplified by FAR’s “step-like” emission surge after 1.0–1.5 and the sky view factor (SVF)’s pronounced L-shaped reduction. (3) Functional heterogeneity determines impact, evidenced by a hybrid morphology-emission mechanism in industrial blocks (IBs) and FAR’s directional reversal in green space (GS). These findings demonstrate that low-carbon planning must shift toward multidimensional management informed by geographic constraints, nonlinear thresholds, and functional zoning.

## Highlights

• Geo-constraints are the primary drivers of street block scale CE intensity
• Nonlinear thresholds and saturation effects govern UM-CE intensity relationships
• A step-like threshold for FAR’s emission effect is quantified at 1.0–1.5
• Explainable ML (XGBoost-SHAP) quantifies complex street block scale drivers

## Graphical abstract

**Figure:**
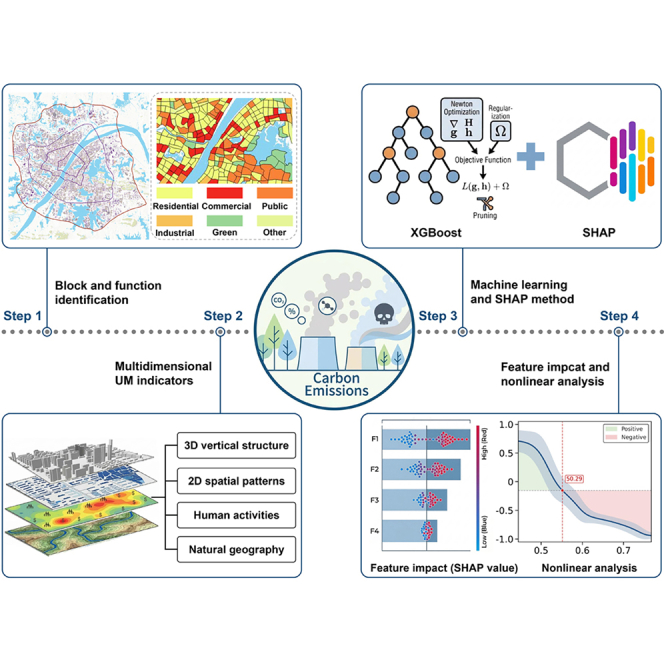

## Introduction

Rapid urbanization and the convergence of populations have enhanced both efficiency and accessibility.^1^^,^^2^ However, cities, which occupy only about 2% of the Earth’s landmass, consume roughly two-thirds of global energy and generate approximately 70% of greenhouse gas emissions.^3^ Carbon dioxide is the dominant anthropogenic greenhouse gas. Consequently, amid the dual challenges of the global low-carbon energy transition and inadequate spatial governance, urban energy demand and carbon emissions (CEs) continue to rise.^4^^,^^5^ In response to the global climate emergency, major economies worldwide have aligned with the Paris Agreement to pursue net-zero targets.^6^ Rapidly urbanizing regions, particularly in the Global South and Asia, face a dual challenge: accommodating population growth while urgently decarbonizing their high-density urban fabrics. Consequently, developing measurable and transferable mitigation pathways for high-density cities globally has become a critical priority.

Although the critical role of urban morphology (UM) in influencing CE is widely acknowledged, significant uncertainty persists regarding the specific response relationships and underlying mechanisms.^7^ High emissions are not an inevitable consequence of urbanization; rather, UM and spatial organization are the core factors determining energy consumption and emission pathways. UM, defined as the spatial pattern and structural characteristics of land use, impacts energy consumption and emissions through multiple pathways, including building-environment coupling, the spatiotemporal organization of activities, and service accessibility.^8^^,^^9^ Compared to macro-level industrial or energy restructuring, optimizing UM offers greater spatial operability and cost-effectiveness. This allows for direct intervention at the implementation scale through planning, architecture, and landscape design.^10^^,^^11^ Without compromising reasonable development goals, morphological optimization has the potential to reduce emissions and improve energy efficiency. Therefore, constrained by the cost bottlenecks of technological abatement and the lag in policy execution, optimizing UM to guide low-carbon behaviors and reduce endogenous energy demand is emerging as a “third path” that is both cost-effective and resilient. This approach provides a crucial spatial lever for urban renewal and sustainable development.

UM represents the geographic spatial manifestation of urban development and residents’ living conditions,^12^ spanning multiple scales, including regions,^13^ cities,^14^ neighborhoods,^15^ and buildings.^16^ In terms of composition, a higher proportion of energy-intensive land use is typically associated with increased emissions, while the contraction of blue-green spaces often corresponds to rising heating and cooling loads.^17^^,^^18^ In terms of pattern, higher patch dispersion and lower network connectivity tend to reduce economies of scale and lengthen necessary travel.^19^ Conversely, moderate agglomeration, higher functional mixing, and continuous blue-green networks are generally associated with shorter activity distances and improved microclimates.^20^ These findings collectively highlight the need to identify and quantify the causal chain between UM and CE within a measurable indicator framework.

Existing research has predominantly focused on urban and regional scales, often comparing total or per capita emissions while concentrating on macro-level issues such as the relationship between urban structure, urban sprawl, and compactness with emissions.^21^^,^^22^ Most evidence suggests that higher compactness and continuity are generally associated with lower emissions.^23^^,^^24^ However, this macro-perspective fails to explain block-level variations and their underlying mechanisms. Specifically, existing research faces three core challenges in finely resolving this relationship: first, a scale mismatch, where macro-level emission inventories do not align with the granularity of micro-level morphological indicators^25^^,^^26^; second, dimensional limitations, with an overemphasis on two-dimensional (2D) patterns while the effects of densification processes and three-dimensional (3D) morphology remain systematically unexamined^27^^,^^28^; and third, a “black box” mechanism, as traditional linear models are ill-equipped to capture the complex nonlinearities and threshold effects governing the relationship.

Recent advances in data and methodologies offer potential pathways to overcome these limitations. The coverage and precision of 3D building data and geographic big data continue to improve, and fine-scale emission proxies and simulations are gradually becoming available.^29^ Building on this, interdisciplinary research has already begun to transition from 2D to multidimensional morphology, accumulating transferable evidence on issues such as urban heat islands (UHIs),^30^ land surface temperatures,^31^ wind environments,^32^ floods,^33^ and urban vitality.^34^ However, research focusing specifically on CE, particularly at the block scale, remains relatively underdeveloped. Few studies have coupled 2D patterns and 3D morphology with CE intensity within a unified framework. Addressing these shortcomings urgently requires data and methods at a finer resolution. The block scale, serving as the minimum common denominator connecting physical mechanisms, statistical identification, and planning practice, is the ideal analytical unit. It avoids the averaging bias inherent at the city or regional level and allows for the identification of operable morphological levers, providing a sound basis for generating robust and interpretable causal evidence.

To address these gaps, we focused on the core area of a representative high-density megacity, Wuhan, as a strategic case study to construct a quantitative framework for analyzing the mechanisms linking multidimensional UM and CE at the block scale. Wuhan exemplifies the rapid expansion and high-density development paradigm common to emerging economies, making it an ideal laboratory to test morphological effects that are relevant to growing metropolitan regions worldwide. The research aims to build and validate an interpretable quantitative framework to reveal the nonlinear mechanisms, thereby providing a scientific basis and actionable pathways for fine-grained, low-carbon urban renewal. The core contributions of this study are 3-fold: (1) In terms of its research paradigm, we establish an integrated morphology-emission analytical framework that expands the research focus from 2D patterns to the coupling of 3D structure and the natural geographic substrate. (2) Methodologically, we integrate fine-grained block partitioning with advanced techniques (XGBoost and SHAP) to open the “black box” of morphological impacts on CE, precisely identifying factor importance, thresholds, and interaction effects. (3) From an application standpoint, we reveal the spatial heterogeneity of these mechanisms through scenario-based assessments, providing actionable, quantifiable, and differentiated morphological optimization pathways for urban planning.

**Figure 1:**
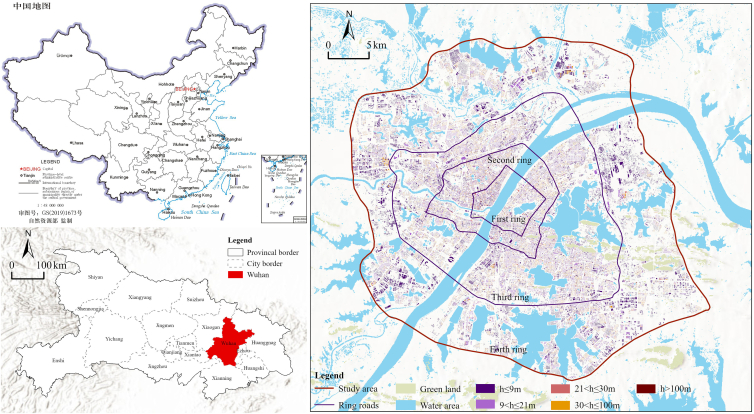
Study area location and characteristics

This study selects Wuhan, a megacity in central China, as a strategic empirical representative of high-density urbanization in the Asia-Pacific region. The city serves as an ideal natural laboratory characterized by a composite urban fabric, where densely populated cores coexist with expanding peripheries. Its structural complexity manifests both vertically, with a diverse building profile ranging from low-rise to skyscrapers (Figure 1), and horizontally, where the built environment is deeply intertwined with an extensive hydrological network. This distinct landscape creates a fragmented urban fabric where subtle topographic changes significantly dictate development feasibility.

## Results

### Research framework

**Figure 2:**
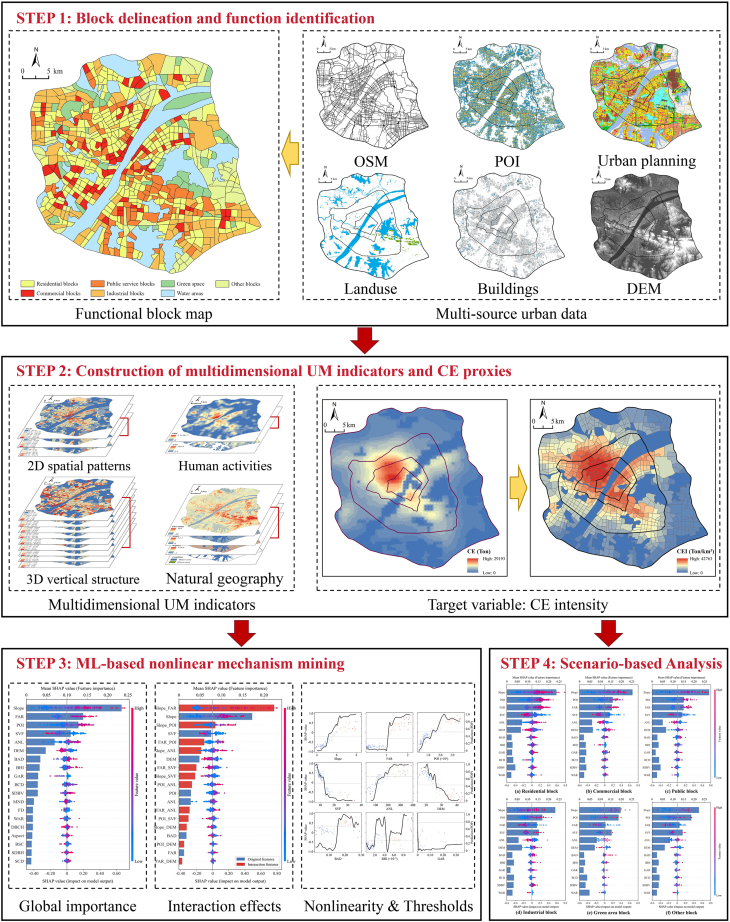
Research framework

This study aims to systematically reveal the nonlinear and spatially heterogeneous relationships between multidimensional UM and CE intensity. We constructed a research framework comprising four core stages: (1) block delineation and functional identification, (2) construction of multidimensional UM indicators and CE proxies, (3) machine learning (ML)-based nonlinear mechanism mining, and (4) heterogeneity assessment across functional scenarios. This research pathway, illustrated in Figure 2, moves from refined spatial unit delineation and indicator quantification to advanced nonlinear modeling and scenario-specific analysis.

### Characteristics of delineated blocks and functions

**Figure 3:**
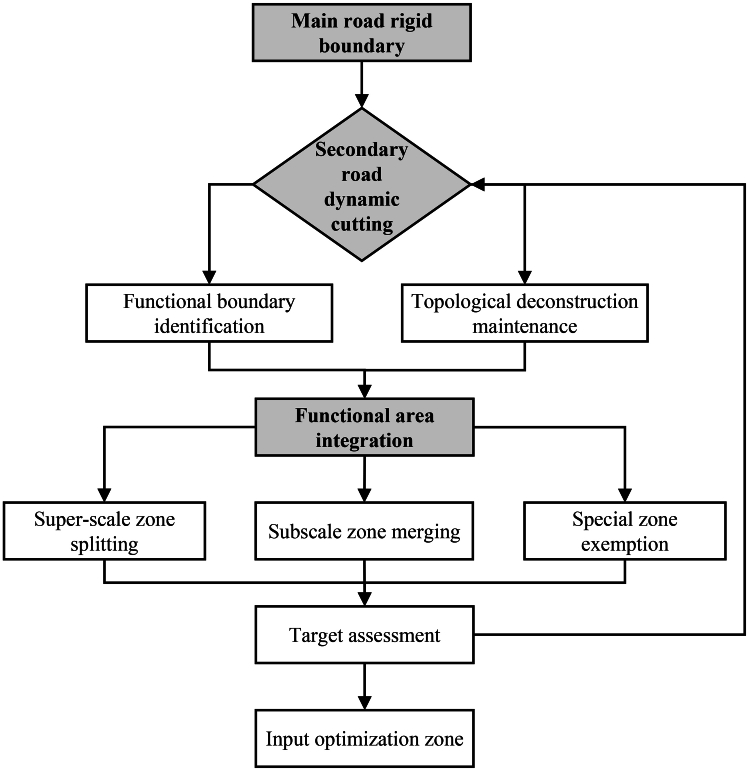
Block delineation flowchart

**Figure 4:**
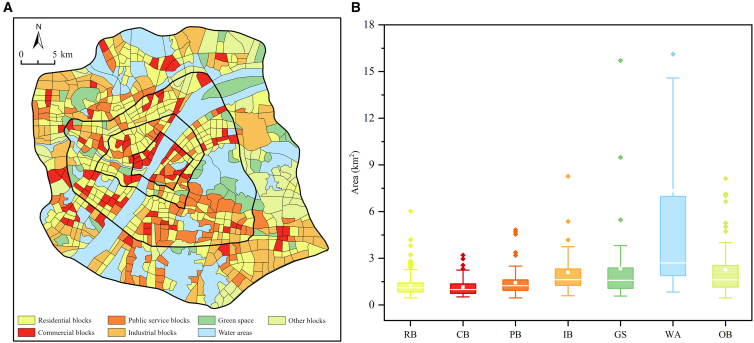
Spatial and functional characteristics of urban blocks (A) Functional zoning map, where colors represent different functional block types. (B) Area distribution by functional block type. In boxplots, the central line indicates the median, the box spans the interquartile range (25th to 75th percentiles), whiskers extend to 1.5 times the interquartile range, inner white squares denote means, and rhombic dots indicate outliers.

Refined spatial delineation yielded 708 functionally homogeneous block units (Figure 3), forming a structured functional landscape (Figure 4A). Residential blocks (RBs) form the underlying matrix, distributed extensively across all precincts. Commercial (CB) and public blocks (PBs) exhibit polycentric clustering along core nodes and transportation corridors, driving urban vitality. In contrast, industrial blocks (IBs) display pronounced peripheral clustering. Green space (GS) is interconnected along river and lake systems, constituting the city’s ecological infrastructure. This pattern provides an ideal setting for examining mechanism heterogeneity.

Functional block types also exhibit significant differentiation in spatial scale (Figure 4B). RB, CB, and PB feature smaller median areas (0.5–3 km^2^), reflecting their intrinsic scale as finely organized units. Conversely, IB includes large-scale parcels (>20 km^2^), consistent with production requirements. This scale differentiation corroborates the observed spatial organization. Water areas (WAs) were excluded from modeling as they are not direct drivers of anthropogenic CE.

### Spatial correspondence between CE intensity and multidimensional UM

**Figure 5:**
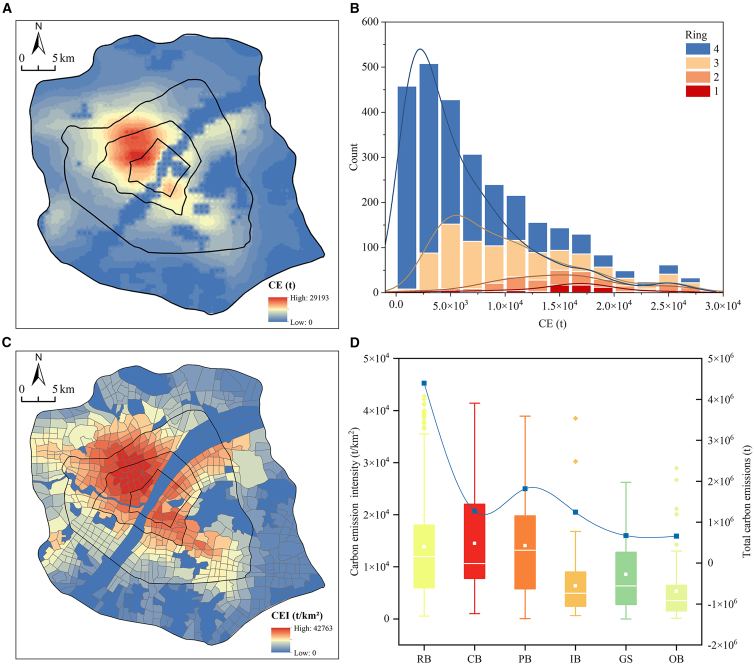
Spatial and functional differentiation of CE (A) Total CE distribution, with red and blue indicating high and low emission values of original rasterized data. (B) Total CE by ring, presented as a histogram color-coded by concentric rings. (C) Block-scale CE intensity, using a blue-to-red gradient for low-to-high aggregated block means. (D) Total CE and intensity by function. In CE intensity boxplots (left y axis), the central line indicates the median, the box spans the interquartile range (25th to 75th percentiles), whiskers extend to 1.5 times the interquartile range, white squares denote means, and dots indicate outliers. The solid blue line with square markers indicates total CE by functional block type (right y axis).

Urban CE exhibits dual differentiation across geographic and functional spaces. Geographically, total urban CE displays a highly concentrated, monocentric pattern (Figure 5A). This structure is quantitatively confirmed by the frequency statistics in Figure 5B, where high-emission cells are predominantly concentrated in the inner rings (Ring 1–2), identifying the urban core as the primary emission source. When aggregated to functional blocks (Figure 5C) to derive CE intensity, this core-periphery structure remains robust, with high-intensity zones (peaking at 42,763 t/km^2^) clustering in the inner rings and riverfront areas.

Functionally, the differentiation is complex and asymmetric (Figure 5D). CB exhibits the highest median intensity and dispersion, identifying high-density commercial activity as the key hotspot driver. In contrast, RB contributes high total emissions but maintains lower intensity due to their extensive spatial footprint, while IB shows moderate intensity despite their peripheral locations. This distinct spatial and functional non-stationarity sets the target for identifying the specific morphological drivers.

To reveal the driving mechanisms, we analyzed the spatial characteristics of UM and their coupling relationships with the observed CE patterns.

**Figure 6:**
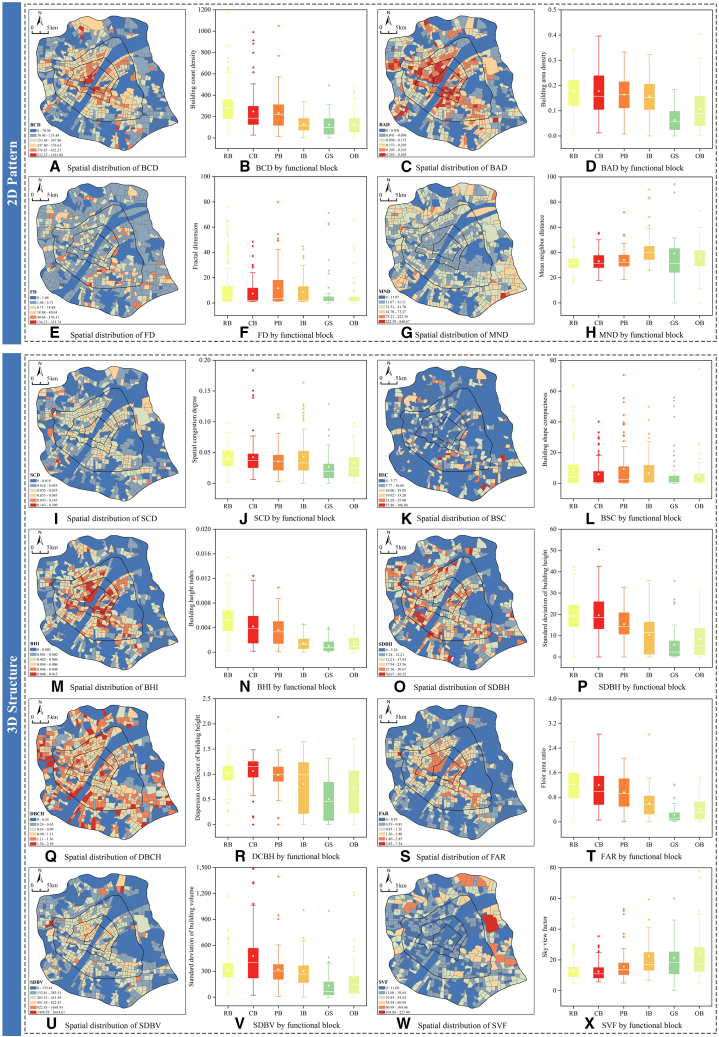
Spatial and functional variation of built environment indicators Left spatial maps show aggregated block-level indicators (blue-to-red gradient: low-to-high values). Data are represented as the mean. In right boxplots, the central line indicates the median, the box spans the interquartile range (25th to 75th percentiles), whiskers extend to 1.5 times the interquartile range, inner white squares denote means, and dots represent outliers.

The built environment constitutes the physical foundation (Figure 6). 2D patterns (building count density, BCD, building area density, BAD) delineate a monocentric structure decreasing from the core, while 3D indicators (floor area ratio, FAR, sky view factor, SVF) reveal that the core area is characterized by high-intensity vertical development and typical street canyon morphologies. Crucially, this steep declining gradient of built intensity is highly coupled with the CE intensity patterns observed in Figure 5C, indicating that vertical urbanization and spatial compaction are the physical prerequisites for emission hotspots.

**Figure 7:**
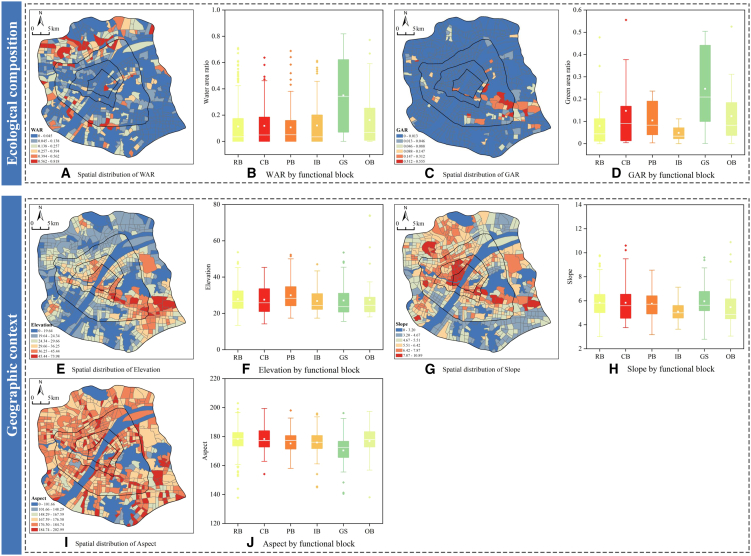
Spatial differentiation of natural environmental elements Left spatial maps show aggregated block-level indicators (blue-to-red gradient: low-to-high values). Data are represented as the mean. In right boxplots, the central line indicates the median, the box spans the interquartile range (25th to 75th percentiles), whiskers extend to 1.5 times the interquartile range, inner white squares denote means, and dots represent outliers.

Natural substrates exert regulatory effects. Ecological patterns (water area ratio (WAR) and green area ratio (GAR)) and topographic features (Figure 7) exhibit a significant inverse spatial coupling with built intensity. The urban core is typically situated in areas with the lowest ecological coverage, lower elevation, and gentler slopes. This reverse distribution suggests that the absence of blue-green spaces in high-density zones may indirectly exacerbate emissions through negative feedback loops.

**Figure 8:**
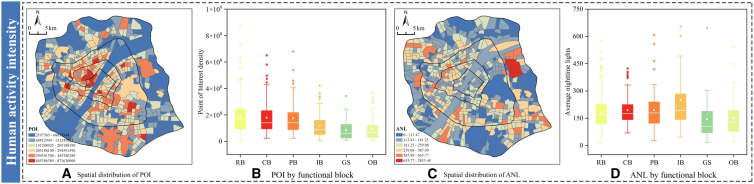
Spatial and functional variation of human activity intensity Left spatial maps show aggregated block-level indicators (blue-to-red gradient: low-to-high values). Data are represented as the mean. In right boxplots, the central line indicates the median, the box spans the interquartile range (25th to 75th percentiles), whiskers extend to 1.5 times the interquartile range, inner white squares denote means, and dots represent outliers.

Human activity intensity (Figure 8) serves as the direct pulse of energy consumption. The spatial distributions of points of interest (POI) density and average nighttime light (ANL) align closely, clearly identifying an activity core composed of CB and PB. This high concentration corresponds directly with the high CE intensity in CB shown in Figure 5D, demonstrating a strong spatial overlap where the agglomeration of socioeconomic functions acts as the direct driving force for emissions.

### Nonlinear and interaction effects of urban morphology based on XGBoost

The XGBoost model, trained with optimized hyperparameters, demonstrated robust predictive performance on an independent test set. The model achieved an R^2^ of 0.77, successfully explaining 77% of the variance in block-level CE intensity. Furthermore, the low RMSE (4627.07) and MAE (3615.28) confirm high consistency between predictions and actual values. Specifically, the Global Moran’s I test on residuals confirmed the effective resolution of spatial dependency, ruling out artifacts of spatial clustering. Moreover, the consistency between training and test set performance demonstrates excellent generalization capability without overfitting. Collectively, these results validate the effectiveness of the ML approach in constructing a high-precision prediction model. To address potential endogeneity risks between the dependent variable and human activity indicators (POI, ANL), we additionally trained a “pure physical form model” as a robustness check. Results demonstrate that this physical-only model maintained robust explanatory power (R^2^ = 0.66), and the global importance of core physical factors, along with their nonlinear response curves, remained consistent. This confirms that the core findings are not spurious correlations arising from common data sources.

**Figure 9:**
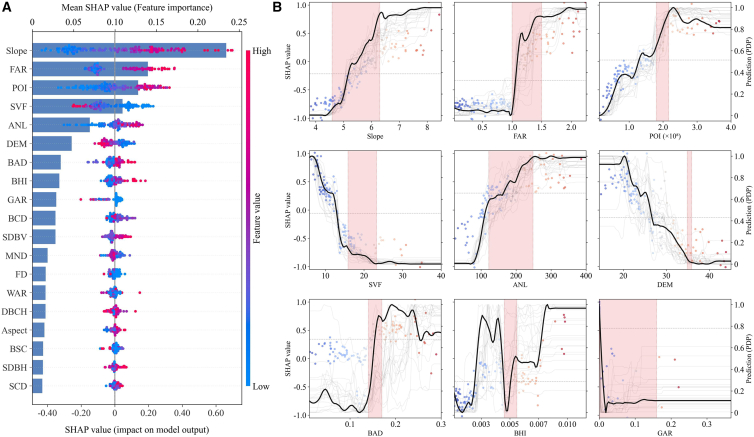
SHAP decomposition of UM’s impact on CE intensity (A) Feature importance and SHAP distribution. Bars show mean absolute SHAP values; beeswarm plots detail individual block distributions (blue to red: low to high feature values). (B) Nonlinear responses of key factors. Gray lines depict 50 retraining iterations; solid black lines represent the robust mean effect. Red shading highlights critical thresholds identified via segmented regression. In all panels, dots represent individual blocks.

To unpack the complex relationship between UM and CE intensity, we employed the SHAP framework for in-depth analysis. As shown in Figure 9A, the global importance ranking reveals that urban CE intensity is a complex outcome driven by coupled effects from the natural substrate, built environment, and human activity. Slope (natural environment) ranks first with a significant advantage, emerging as the primary predictor. This confirms the fundamental constraints that geographic context imposes on urban development effects. Following closely are FAR, POI, SVF, and ANL, comprehensively covering the three core dimensions. Secondary factors such as elevation, BAD, and building height index (BHI) also hold significant positions, validating the necessity of the integrated analytical framework.

Beyond global ranking, dependency plots (Figure 9B) reveal that the environmental effects of morphological factors are characterized by pronounced threshold and saturation behaviors. As the primary factor, the emission-increasing effect of slope is concentrated within a sensitive range (4°–6.5°), rapidly saturating thereafter. The 3D built form reflects complex trade-offs. FAR exhibits a distinct “step-like” pattern: its contribution to emissions surges sharply after crossing the threshold range between 1.0 and 1.5. SVF exhibits a strong inverse effect, where low values (15) correlate closely with high CE intensity, while emission-reducing benefits emerge rapidly and plateau as SVF increases. Complementing this, BAD triggers distinct emission surges once coverage exceeds 0.15. Human activity intensity (POI and ANL) also shows the most pronounced growth in low-value ranges. This dominant mechanism is modulated by other variables; for instance, elevation exhibits clear negative effects that stabilize beyond 27 m, while FAR provides synergistic emission-reduction pathways with diminishing marginal returns. These complex nonlinear relationships indicate that universal morphological optimization strategies are inefficient, and future planning must be grounded in identifying and acting upon these sensitive intervals.

**Figure 10:**
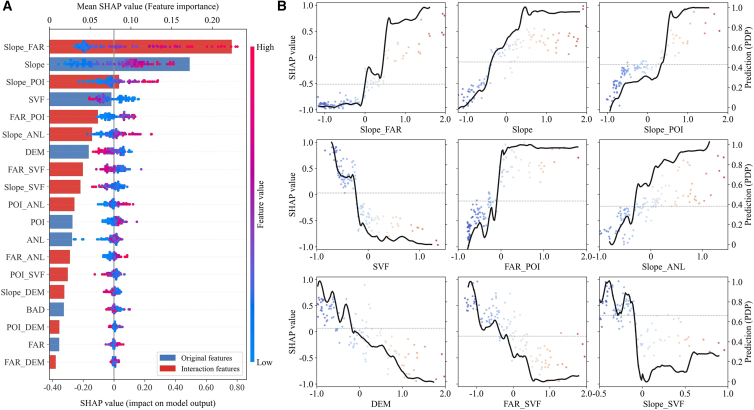
SHAP interaction effects on CE intensity (A) Main and interaction effect ranking. Bars show mean absolute SHAP values (blue: original features; red: interaction features); beeswarm plots detail individual block distributions (blue to red: low to high feature values). (B) Dependency of key interactions. Solid black lines represent the average prediction trend. In all panels, dots represent individual blocks.

While the preceding analysis revealed individual pathways, cities are complex coupled systems where elements do not operate in isolation. We employed SHAP interaction values to uncover synergistic and antagonistic relationships. Interaction effects are comparable in importance to—and in some cases surpass—main effects. As shown in Figure 10A, Slope_FAR emerges as the most significant interaction, ranking first globally. Similarly, terms like Slope_POI and FAR_POI occupy top positions, demonstrating that ignoring coupling relationships leads to an incomplete understanding of drivers. These interactions primarily manifest in two patterns: “geographic background–development intensity” and “development intensity–activity intensity,” suggesting profound synergistic effects within the site selection-development chain.

Dependency plot analysis reveals specific underlying mechanisms. The interaction between slope and FAR exhibits a significant positive correlation (Figure 10B). This reveals a mechanism where the geographic substrate influences development patterns: flat terrain serves as both a prerequisite and an amplifier for ultra-high-density development. Only when high density occurs on flat terrain can megacity cores form, creating a synergistic “1 + 1>2” emission increase. Conditional partial dependence analysis quantifies this surge, revealing that on flat terrain (Slope ≤5°), increasing FAR from 1.0 to 2.0 corresponds to a disproportionate emission increase of 24.9%. Conversely, the interaction between FAR and SVF reveals a classic planning trade-off. The overall negative correlation indicates that the emission-increasing effect of FAR is counteracted by the emission-reducing effect of SVF. Specifically, blocks with the extreme combination of high FAR and low SVF show the highest CE intensity, reflecting the negative effects of congested built environments. This suggests that in high-density contexts, optimizing building layout to maintain spatial openness (high SVF) is an effective pathway to counteract environmental impacts. Crucially, under scenarios where FAR >1.5, this strategy yields a carbon intensity reduction of 31.1% as SVF increases from 7.3 to 22.5, providing a quantitative basis for compensatory zoning.

### Heterogeneity of driving mechanisms across functional zones

**Figure 11:**
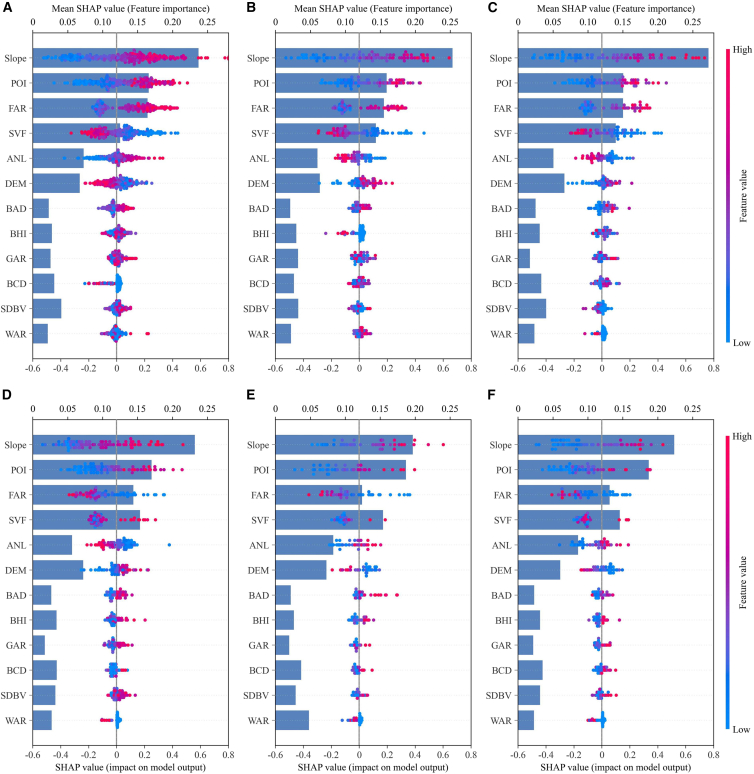
Heterogeneity of CE intensity drivers by functional block (A) Residential block. (B) Commercial block. (C) Public block. (D) Industrial block. (E) Green space. (F) Other block. In all panels, bars show mean absolute SHAP values; beeswarm plots detail individual block distributions (blue to red: low to high feature values). Dots represent individual blocks.

To investigate driving mechanisms across distinct functions, we performed a conditional analysis of the globally trained model. Results reveal a differentiation of driving patterns (Figure 11), transitioning from universal principles to function-specific scenarios. Within primary functional blocks (RB, CB, PB), the ranking largely aligns with the global pattern, reflecting the universal principle that built density and human activity serve as core drivers. However, the model captures a localized amplification effect: in CB and PB, the influence of POI density is significantly amplified, with SHAP values in high-density areas concentrated in the strong positive range. This indicates that intensive energy demands in commercial environments specifically amplify the emission-increasing effect of activity intensity.

A more profound heterogeneity manifests in specific scenarios. In IB, driving patterns exhibit a complex “Geo-Functional” constraint. Slope ranks first, reflecting the rigid topographic requirements for industrial siting. Crucially, 3D morphology (FAR) remains a top-tier driver alongside facility density (POI), yet its influence tends to saturate, indicating that emissions are determined by the coupling of construction scale and operational intensity. This mixed driving pattern likely reflects the internal heterogeneity of industrial activities, encompassing both morphology-sensitive and process-driven sectors. Another anomaly occurs in GS, where FAR exhibits a directional reversal, showing a significant negative correlation. Structural verification substantiates the mechanism behind this trend: unlike residential zones, FAR in GS is strongly correlated with vertical height (r = 0.69) while building density remains strictly limited (mean 7.4%). The evidence validates vertical consolidation as a superior alternative to low-density sprawl, limiting ecological encroachment to preserve carbon sinks and optimize net emissions.

**Figure 12:**
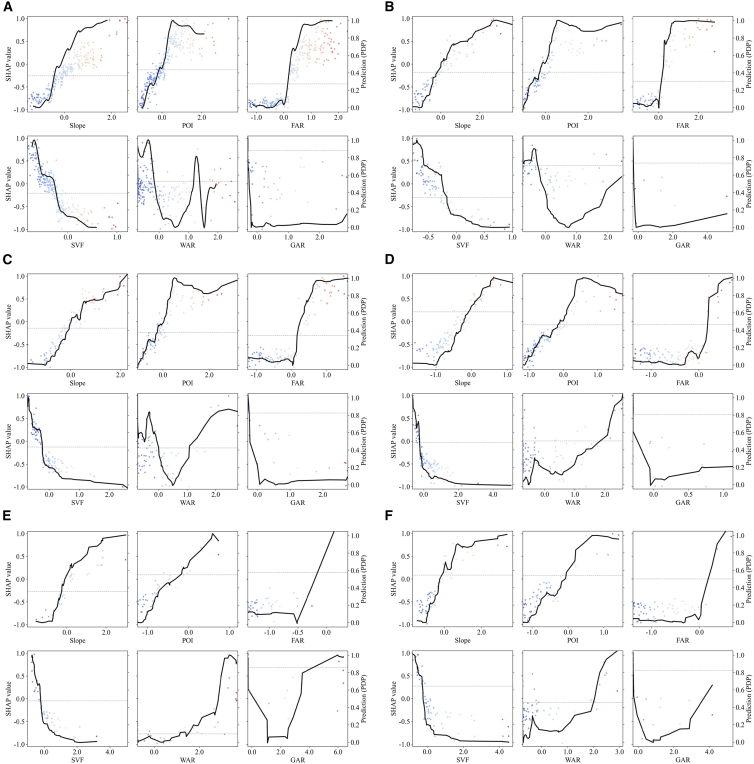
Nonlinear effects of key drivers by functional block (A) Residential block. (B) Commercial block. (C) Public block. (D) Industrial block. (E) Green space. (F) Other block. In all panels, solid black lines represent the average prediction trend; dots represent individual block distributions (blue to red: low to high feature values).

To further elucidate these functional differences, we analyzed the nonlinear response curves for six key indicators (Figure 12). These curves reveal that driving relationships are not simple linear trends but contain critical thresholds and saturation intervals.

In human-dominated RB (Figure 12A), CB (Figure 12B), and PB (Figure 12C), dominant indicators like POI and FAR generally exhibit strong positive driving forces with localized variations. Notably, in CB, the influence of POI surges sharply from extremely low values with virtually no buffer period, reflecting the immediate, high-intensity energy consumption of commercial activities. In contrast, the POI effect in RB exhibits a typical S-shaped curve, entering a rapid growth phase only after surpassing a certain threshold. Similarly, SVF demonstrates a negative inhibitory effect across all three functional blocks, with the most pronounced impact observed in its low-value range, confirming the emission-reduction benefits of preserving open spaces within high-density environments.

For IB, the response curves corroborate the distinct “Geo-Functional” constraint mechanism. Specifically, the FAR curve (Figure 12D) displays a clear positive stepwise increase, confirming that physical construction intensity remains a structural driver of emissions in industrial zones, mirroring the steep positive response observed for Slope. In contrast, GS presents a distinct “inverse” pattern (Figure 12E). Here, the FAR curve exhibits a directional reversal, dipping into negative SHAP values within specific ranges. This phenomenon quantitatively reflects the ecology-construction trade-off, where consolidated development patterns effectively mitigate disturbances to carbon sinks compared to dispersed layouts.

## Discussion

### Nonlinearity and thresholds of multidimensional drivers

Although our study corroborates the built environment’s dominant role, a critical discrepancy emerges regarding dimensionality. Unlike traditional research focusing on planar metrics,^24^ our global ranking identifies 3D indicators (FAR, SVF) as superior predictors. This suggests that emissions are inherently volumetric; unlike 2D metrics, 3D indicators serve as accurate proxies for the total volume of energy-demanding activities and vertical heat dissipation capacity. This distinction provides evidence supporting calls to expand morphology research to 3D.^35^^,^^36^ Additionally, distinct from studies treating topography as a static background, our model reveals slope as an antecedent determinant in riparian environments. It actively constrains infrastructure efficiency and road tortuosity before construction even begins, thereby setting the physical baseline for regional carbon intensity.

Our framework challenges the linear assumptions in conventional studies.^20^ Contradicting the compact city theory’s straightforward density-emissions correlation, our results expose a distinct step-like surge in FAR (1.0–1.5) rather than continuous efficiency gains. This marks a critical typological transition from low-rise to multi-story fabrics,^37^ signaling a nonlinear accumulation of infrastructure baseloads. Thermodynamically, this interval represents where street canyon effects impede natural ventilation,^38^ implying a regime shift from passive cooling to energy-intensive HVAC systems. Thus, morphological compactness ceases to be an efficiency asset and becomes a thermodynamic liability.

These insights refine the density-efficiency debate,^21^ suggesting compaction benefits are bounded by physical limits. Consequently, zoning must shift to differentiated strategies aligned with these thresholds: the FAR 1.0–1.5 interval should function as a conditional permitting zone requiring climatic assessments, while 1.5 serves as a strict regulatory boundary. Beyond this point, density increases must be coupled with compulsory ventilation requirements (minimum SVF), dynamically balancing accommodation capacity with thermodynamic limits.

### Geographic constraints and morphological trade-offs

Challenging the prevailing tendency to focus on anthropogenic factors,^7^^,^^19^ slope emerged as the primary driver, functioning primarily as an “Antecedent Geographic Constraint.” Unlike direct operational predictors, its influence is indirect, mediated by locking in development suitability and infrastructure efficiency before the built environment is even established.

This mechanism aligns with urbanization patterns in global deltaic regions like Amsterdam or the Pearl River Delta,^39^^,^^40^ yet Wuhan presents a distinct “sensitive threshold” mechanism. Unlike mountainous cities where slope acts as a continuous barrier,^41^ or uniform coastal deltas,^42^ slight topographic variations here drive carbon intensity through three interlinked pathways. Geotechnically, flat terrain minimizes foundation risks in soft-soil zones, serving as a prerequisite for ultra-high-density clusters.^43^ Morphologically, it facilitates orthogonal road grids that concentrate vehicular flows, contrasting with the tortuous layouts necessitated by undulating terrain.^44^ Microclimatically, low-lying basins entrap heat and exacerbate cooling loads, whereas sloped terrains benefit from superior natural ventilation.^45^ Thus, terrain acts not merely as a passive backdrop but as an active biophysical determinant constraining the spatial capacity for carbon-intensive activities.

This foundational role is amplified by interactive effects, specifically Slope_FAR, which reveals a synergistic “1 + 1>2” risk. Our conditional analysis confirms that on flat terrain, increasing development intensity (FAR from 1.0 to 2.0) yields a disproportionate emission surge of 24.9%, validating the amplifier hypothesis. Conversely, morphological design offers optimization space via the FAR-SVF antagonism. The impact of high FAR is strongly modulated by SVF: low SVF exacerbates UHI effects and cooling loads,^31^^,^^46^ whereas optimizing spatial openness significantly mitigates this. Physical validation using high-resolution LST data identifies a significant positive correlation in deep street canyons, confirming distinct daytime shading benefits that contrast with the ventilation-driven accumulation of carbon intensity. Crucially, simulation demonstrates that increasing SVF (from 7.3 to 22.5) under high-density scenarios yields a potential carbon intensity reduction of 31.1%. This necessitates a “slope-indexed density control” mechanism: in flat, high-risk zones, ultra-high FAR permits must be capped or require mandatory open-space compensation (high SVF) to offset the density-induced carbon premium, while complex terrain requires strict footprint limits to minimize ecological disturbance.

### Functional heterogeneity of CE intensity mechanisms

Exposing functional heterogeneity often obscured by uniform approaches,^22^ our model reveals that human-dominated sectors (RB, CB, PB) exhibit divergent sensitivities despite a shared positive driving pattern. While CB and PB act as emission amplifiers due to high baseloads, RB displays a saturation behavior. Consistent with nonlinear transport studies,^47^ RB benefits reverse beyond specific saturation points, indicating a traffic-induced rebound effect from excessive commercialization.^48^^,^^49^

In IB, a hybrid mechanism emerges where morphological constraints drive emissions alongside functional intensity. Here, the prominence of ANL captures the dynamic pulse of operational vitality and economic throughput, indicating that emissions are fueled as much by the metabolic pace of production as by the physical scale of facilities. This implies the sector is not a monolith of form-neutral industries but encompasses activities where spatial configuration remains a constraint.^50^ For GS, we observed a counterintuitive reversal of the density effect. Structural verification supports this, revealing that FAR in GS is driven by vertical height (r = 0.69) rather than footprint (mean density 7.4%). Consequently, high-density consolidation of essential facilities yields superior net carbon performance, aligning with the land sparing hypothesis,^51^ by minimizing encroachment on the ecological matrix.^52^

Based on these diagnostics, we propose a differentiated density allocation framework. For human-dominated sectors, planning must shift from maximizing mix to regulating intensity thresholds, zoning overlays in RB should cap commercial-to-residential ratios at inflection points to prevent rebound effects,^53^^,^^54^ while CB and PB require energy efficiency standards to decouple density from emissions.^55^ For IB, hybrid drivers necessitate operational-based controls superseding pure morphological metrics. Regulations should prioritize regulating metabolic intensity while restricting land-intensive manufacturing to flat terrain to prevent infrastructure lock-in.^56^ For GS, the observed density reversal supports a conservation-through-compaction strategy: regulations should enforce minimum floor area ratios for essential facilities to compel vertical consolidation, maximizing the contiguous ecological matrix for carbon sequestration.^57^

In summary, this study clarifies the mechanisms linking urban morphology to carbon intensity. We demonstrate that this relationship goes beyond simple linear assumptions. Instead, urban CE is an inherently volumetric process driven by the natural terrain, the built environment, and human activity. Our findings establish a replicable framework for fine-scale analysis by highlighting nonlinear thresholds, interactive constraints, and function-specific behaviors. Consequently, our results challenge the reliance on uniform density controls. Urban planning must transition to differentiated strategies that explicitly account for geographic constraints. Practical policy shifts are therefore necessary. High-risk terrains require stricter floor area ratio limits. Furthermore, performance-based zoning should couple density allowances with spatial openness. Diverse functional sectors also need tailored regulations that address both operational and morphological factors. Although simulation scale constraints remain, these empirical insights provide a robust foundation for advancing targeted and scientifically grounded low-carbon urban design.

### Limitations of the study

Despite these findings, limitations remain. Regarding the carbon proxy, the 1 km ODIAC data introduces inherent uncertainties regarding sub-grid heterogeneity, although the area-weighted method statistically supports macro-scale gradient identification. Methodologically, this cross-sectional design identifies spatial associations rather than confirmed temporal causality; thus, the results reflect morphological equilibrium rather than dynamic evolution. Consequently, longitudinal studies are required to test threshold robustness over time. Furthermore, the dominant role of Slope is likely context-dependent, specifically characterizing riparian flatlands, and necessitates comparative verification in mountainous or arid regions. Future efforts should also integrate building physics (age, materials) and resident attributes to develop a unified framework.

## Resource availability

### Lead contact

Requests for further information and resources should be directed to and will be fulfilled by the lead contact, Gangyi Tan (tangangyi_hust@163.com).

### Materials availability

This study did not generate new unique reagents.

### Data and code availability

• All relevant materials, including the underlying model code and the multidimensional urban indicator datasets utilized in this research, have been deposited in the Mendeley Data repository and are openly accessible via: https://data.mendeley.com/datasets/n9ptzv2fzb/1
• All custom code is available on request from the lead contact.
• Any additional information required to reanalyze the data reported in this paper is available from the lead contact upon request.

## Acknowledgments

We would like to express our sincere gratitude to the editors and reviewers who have put considerable time and effort into their comments on this paper. This work was supported by a grant from the 10.13039/501100001809National Natural Science Foundation of China (52278018) and the National Key Research and Development Program of China (2024YFC3808300).

## Author contributions

Conceptualization, J.Z., Z.C., and G.T.; methodology, J.Z. and Z.C.; investigation, J.Z. and Z.C; writing – original draft, J.Z. and Z.C; writing—review and editing, J.Z., Z.C., and G.T.; funding acquisition, G.T.; resources, J.Z. and G.T.; supervision, J.Z., Z.C., and G.T.

## Declaration of interests

The authors declare no competing interests.

## STAR★Methods

### Key resources table

**Table:** 

REAGENT or RESOURCE	SOURCE	IDENTIFIER
**Deposited data**		

Road network (Vector, 2023)	Open Street Map (OSM)	https://www.openstreetmap.org/
Administrative boundary (Vector, 2024)	Resource and Environmental Science Data Platform (RESDC)	https://www.resdc.cn/
Land use (Raster, 30 m × 30 m, 2023)		
Digital Elevation Models (DEM) (Raster, 30 m × 30 m)		
Nighttime lighting (Raster, 500 m × 500 m, 2020)		
Building data (Vector, 2023)	Baidu map API	https://www.baidu.com/
POI (Vector, 2023)		
Urban planning map (Vector, 2025)	Wuhan City Platform for Common GeoSpatial information Services	https://hubei.tianditu.gov.cn/
Monthly CO^2^ gridded emission (Raster, 1 km × 1 km, 2023)	Open-Data Inventory for Anthropogenic CO^2^ (ODIAC)	https://db.cger.nies.go.jp/
Raw data and optimized code	This paper	https://data.mendeley.com/datasets/n9ptzv2fzb/1

**Software and algorithms**		

ArcGIS 10.8	ESRI	https://desktop.arcgis.com/
python 3.9.13	python	https://www.python.org/

### Method details

#### Study area and data sources

The study focuses on the core development zone enclosed by the Fourth Ring Road (approximately 1,278 km^2^) (Figure 1). Within this scope, we delineated 708 geographic blocks as fundamental analytical units to ensure relative spatial homogeneity. These blocks serve not only as carriers for aggregating multidimensional indicators and block-level CE intensity but also provide a robust foundation for the subsequent detailed analysis of morphological effects and spatial interventions.

The study integrates multi-source datasets encompassing carbon emissions, 3D building morphology, road networks, and socioeconomic attributes. The core carbon emission data is sourced from the ODIAC, a 1 km resolution global product selected for its ability to characterize spatial heterogeneity in high-density contexts. Morphological metrics were derived from 3D building data (footprints and floors) obtained via the Baidu Maps API, road networks from OSM, and terrain variables from DEM. Land use and GDP data were also incorporated to enhance the model’s explanatory power. Although minor temporal inconsistencies exist among datasets (using CE 2023 data), the high short-term stability of the urban core’s physical structure ensures that the identified structural correlations remain robust. All data were uniformly aggregated to each block unit and standardized to the WGS_1984_UTM_Zone_50N coordinate system.

#### Urban block delineation and functional identification

##### Urban block delineation method

Defining an appropriate analytical unit is fundamental to granular UM research. Traditional units, such as administrative boundaries or regular grids, often fail to align with the city’s actual morphological structure and functional organization. The former have arbitrary boundaries, while the latter fragments the coherent urban fabric and introduces severe Modifiable Areal Unit Problem (MAUP).^31^^,^^58^ To overcome these limitations, this study adopts the concepts of geographic entities and functional zones to develop a block delineation method based on the dual constraints of road network-functional block system.^59^^,^^60^ This approach generates analytical units that better reflect the functional-morphological unity of cities.

This delineation process primarily consists of three stages (Figure 3). First, road network-based skeleton segmentation establishes the main road rigid boundary as the unit-delimiting framework, using the expressway and arterial and collector road networks from OSM. These high-level roads serve as the most prominent physical barriers between urban precincts, and initial geometric segmentation along these roads generates foundational block units at the macro-level. Second, function-based internal optimization performs a secondary subdivision using land use data to enhance functional homogeneity. This process refines macro-blocks containing multiple functional parcels by identifying the contours of actual functional zones, ensuring high functional consistency in the final units. Finally, scale-based unit consolidation merges excessively small parcels resulting from road network fragmentation while exempting the boundaries of special functional zones to preserve their geographic integrity.

The 708 urban blocks generated through this process serve as the fundamental analytical units for this study, offering multiple advantages. Defined by the actual road network and internal functional refinement, these units ensure both morphological integrity and a high degree of functional homogeneity. Furthermore, this method achieves superior scale compatibility, generating units of diverse scales that can be effectively matched with data from various sources.^61^^,^^62^ This characteristic is particularly crucial for aggregating the 1 km resolution CE data, as it mitigates the MAUP more effectively than traditional regular grids and establishes a robust spatial foundation for constructing a reliable dependent variable.

##### Dominant function-based block classification

Building on the geometric delineation, we developed a block-level classification system to assign functional attributes. Acknowledging the prevalence of mixed-use fabrics in high-density cities, this study adopts a “Dominant Function Identification” strategy. The identification process involves three steps: (1) Dominant function determination: We used spatial overlay analysis to calculate functional area proportions based on planning data, identifying the dominant function with a 60% threshold.^63^ (2) Continuity verification: We checked spatially adjacent blocks to correct illogical discontinuities based on planning logic. (3) POI-based mixed-use refinement: For transition zones or complex parcels where static planning data creates ambiguity, we analyzed the functional composition of POIs. Specifically, we calculated the ratio of POIs belonging to different functional categories to identify the *de facto* dominant function. This approach allows for a more accurate classification of mixed-use areas by capturing the prevailing socioeconomic attribute rather than merely the physical land use.^64^^,^^65^

#### Multidimensional indicator quantification and variable definition

##### Multidimensional UM indicator system

To assess the complex influence of UM on CE, this study constructs a comprehensive indicator system across three dimensions: built environment morphology, natural environmental elements, and human activity intensity.^66^ This framework treats the city as a complex system of interacting architecture, ecology, and socioeconomic activities, thereby capturing CE drivers more comprehensively (Multidimensional UM indicator framework).

The built environment serves as the decisive physical foundation influencing energy efficiency. For 2D spatial patterns, we extracted indicators including BCD, BAD,^67^ and fractal dimension (FD)^68^^,^^69^ to quantify planar agglomeration, land coverage intensity, and morphological complexity. These metrics collectively reflect urban compactness, directly influencing transport-related CE. For 3D vertical structure, we calculated indicators such as FAR, BHI,^36^ and SVF to characterize vertical development intensity and street canyon effects. These 3D morphologies directly influence building operational CE by affecting microclimate and solar access.

Natural environmental elements play a crucial regulatory role in urban carbon cycles and local climate. For ecological configuration, we introduce WAR and GAR to assess the carbon sink potential of blue-green spaces and their mitigation effects on UHI,^70^ which in turn indirectly reduces building cooling load. For geographic context, we integrate topographic indicators such as elevation, slope, and aspect. Specifically, slope is defined here as a proxy for “geo-environmental constraints” to capture its antecedent limits on construction suitability. Collectively, these elements form the physical foundation of UM, acting as constraints on urban expansion patterns and energy service system layouts.

Human activity intensity reflects the spatial concentration and energy use characteristics of socioeconomic activities, serving as the most direct driver of urban energy consumption and CE. We employ POI density to measure functional facility concentration and combine it with average nighttime light (ANL) to comprehensively quantify human activity intensity. These indicators directly map the energy consumption patterns driven by human socioeconomic activities and are core drivers of urban CE.

**Table:** Multidimensional um indicator framework

Category	Name	Calculation Formula	Description
2D spatial patterns	Building count density (BCD)	$BCD=\frac{n}{S_{B}}$	Density of building units within the block.
	Building area density (BAD)	$BAD=\frac{1}{S_{B}}\sum_{i=1}^{n}A_{i}$	Building footprint coverage ratio.
	Fractal dimension (FD)	$\text{FD}=\frac{1}{n}\times \sum_{i=1}^{n}{\left(P_{i}/4\right)}^{2}/A_{i}$	Morphological irregularity of building footprints.
	Mean neighbor distance (MND)	$\text{MND}=\frac{1}{n}\times \sum_{i=1}^{N}\left(d_{j}\right)$	Average distance among buildings; indicates layout dispersion.
3D vertical structure	Building shape compactness (BSC)	$\text{BSC}=\frac{1}{n}\times \sum_{i=1}^{n}\left(P_{i}\times H_{i}+A_{i}\right)/V_{i}$	Inverse measure of 3D form compactness
	Spatial congestion degree (SCD)	$\text{SCD}=\frac{1}{H_{\max}\times S_{B}}\times \sum_{i=1}^{n}V_{i}$	3D spatial filling rate
	Building height index (BHI)	$BHI=\frac{1}{S_{B}}\sum_{i=1}^{n}H_{i}$	Cumulative building height relative to block area.
	Standard deviation of building height (SDBH)	$\text{SDBH}=\sqrt{\frac{{\sum_{i=1}^{n}\left(H_{i}-\bar{H}\right)}^{2}}{n}}$	Statistical dispersion of building heights.
	Dispersion coefficient of building height (DCBH)	$\text{DCBH}=\text{SDBH}/\bar{H}$	Dimensionless building height variation.
	Floor area ratio (FAR)	$FAR=\frac{1}{S_{B}}\sum_{i=1}^{n}A_{i}\times F_{i}$	Overall 3D development intensity.
	Standard deviation of building volume (SDBV)	$\text{SDBV}=\sqrt{\frac{{\sum_{i=1}^{n}\left(V_{i}-\bar{V}\right)}^{2}}{S_{B}}}$	Statistical dispersion of building volumes.
	Sky view factor (SVF)	SVF = S_Sky_/S_Img_	Measure of spatial openness.
Ecological elements	Water area ratio (WAR)	WAR = S_Water_/*S*_*B*_	Proportion of water body coverage.
	Green area ratio (GAR)	GAR = S_Green_/*S*_*B*_	Proportion of green space coverage.
Geographic elements	Elevation	Avg(DEM)	Average altitude of the block.
	Slope	Avg(Slope(DEM))	Average surface steepness of the block.
	Aspect	Avg(Aspect(DEM))	Compass direction of the terrain’s slope.
Human activity intensity	Point of interest density (POI)	POI = D_poi_/*S*_*B*_	Density of functional facilities.
	Average nighttime light (ANL)	ANL = V_NL_/*S*_*B*_	Intensity of nighttime light.

*S*_*B*_ is the block area; n is the number of buildings; *A*_*i*_ is the base area of building; *P*_*i*_ is the perimeter of building’s base; *H*_*i*_ is the height of each building; H_*max*_ is the maximum building height; $\bar{H}$ is the average building height; *F*_*i*_ is the number of floors for building; *V*_*i*_ is the volume of building; $\bar{V}$ is the average building volume; S_Img_ is the area of street view images; S_Sky_ is the sky area in street view images; S_Green_ is the green space area; D_poi_ is the number of POI within the block; V_NL_ is the sum of nighttime light values within the block.

#### CE intensity proxy estimation and validation

This study focuses on the influence of UM on the spatial patterns of CE, rather than conducting precise carbon audits for each block.^26^ Given the widespread absence of block-level emission inventories, we constructed spatial proxy indicators based on the global high-resolution inventory ODIAC. While applying 1 km resolution data to finer block units introduces uncertainty, ODIAC is well-validated for capturing relative spatial variations driven by high-intensity human activity.^71^^,^^72^^,^^73^^,^^74^ A study in Shanghai similarly confirms that the ODIAC inventory outperforms other data sources in densely populated central urban districts.^13^ Therefore, the CE intensity constructed here serves as a proxy characterizing the spatial patterns of carbon-intensive activities.

Spatial aggregation of gridded data to socioeconomic units is standard analytical practice.^75^ We employed area-weighted averaging (AWA) to aggregate data to the 708 block units based on spatial overlap. Crucially, the scale-compatible block delineation mitigates aggregation errors; unlike arbitrary regular grids, the “road network-functional zones” approach ensures relative internal functional homogeneity. This aligns with global population mapping methodologies that leverage geographic entities to constrain spatial allocation and minimize signal distortion.^76^ To validate the proxy, we conducted a spatial correlation test against independent 1 km GDP grid data aggregated using the same AWA method. The resulting highly significant positive correlation robustly confirms the reliability of CE intensity in characterizing carbon-intensive economic activities. Thus, the CE intensity represents a rigorously processed and validated proxy suitable for investigating complex nonlinear relationships.^77^

#### XGBoost-SHAP nonlinear modeling framework

To interpret the “black box” mechanism of linear models, we constructed an analytical framework based on explainable ML to mine nonlinear impacts, interactions, and scenario dependencies.^78^

#### Coupled UM and CE prediction model

We employed XGBoost to capture the high-dimensional, nonlinear morphology-emission relationship. XGBoost integrates decision trees via additive training to model complex relationships.^79^ The final prediction function ${\hat{y}}_{i}$ is expressed as:

Equation 1 $${\hat{y}}_{i}=\sum_{k=1}^{K}f_{k}\left(x_{i}\right)$$

where *x*_*i*_ is the feature vector for the i-th block and *f*_*k*_ is the prediction result of the k-th tree. The model iteratively optimizes *f*_*k*_ by minimizing a regularized loss function L^(t)^ to control complexity and improve generalization. The regularized loss function L^(t)^ is defined as:

Equation 2 $$L^{\left(t\right)}=\sum_{i=1}^{n}l\left(y_{i},{\hat{y}}_{i}^{\left(t-1\right)}+f_{t}\left(x_{i}\right)\right)+\Omega \left(f_{t}\right)$$

where *y*_*i*_ is the true CE intensity for the i-th block, ${\hat{y}}_{i}^{\left(t-1\right)}$ is the model prediction from iteration (t-1), *l* is the loss function, Ω(*f*_*t*_) is the regularization term, and *n* is the total number of blocks. The model is optimized using a second-order Taylor approximation of L^(t)^.

To ensure model robustness and reproducibility,^80^ we normalized input features and split the dataset into training (80%) and testing (20%) sets with a fixed random seed. Hyperparameters were optimized using Particle Swarm Optimization (PSO) combined with 10-fold cross-validation exclusively on the training set to prevent data leakage.^81^ The model was then retrained on the full training set using optimal parameters. Generalization performance was evaluated on the independent test set using R-squared (R^2^), Root Mean Squared Error (RMSE), and Mean Absolute Error (MAE), providing a reliable foundation for mechanism interpretation.

#### Driver contribution and attribution

Furthermore, to overcome the inherent “black box” problem of ensemble models, we introduced the SHAP framework.^82^ Based on cooperative game theory, the SHAP method decomposes a prediction ŷi into the sum of contributions from each feature. The SHAP value ϕ_*j*_(*x*_*i*_)is defined as the average marginal contribution of the j-th feature to the prediction, i.e., the change from the base value $E\left[\hat{y}\right]$ to the final prediction ${\hat{y}}_{i}$.

Equation 3 $${\hat{y}}_{i}=E\left[\hat{y}\right]+\sum_{j=1}^{M}\phi_{j}\left(x_{i}\right)$$

where *M* is the total number of features, and ϕ_*j*_(*x*_*i*_) is the SHAP value of the j-th morphological feature for the i-th block’s prediction. The mean SHAP values can be used to quantify the global importance ranking of all morphological features, thereby identifying the dominant drivers.

#### Interaction and threshold analysis

To deeply mine the complex mechanisms from the “black box” model, this study examines nonlinear main effects and feature interactions using the SHAP framework. For nonlinear response and threshold identification, we employed a composite visualization method integrating SHAP dependency plots with Partial Dependence Plots (PDP). This method overlays SHAP dependence scatterplots (left axis), reflecting individual sample heterogeneity, with PDP curves (right axis), representing global average trends. This approach allows for the robust identification of nonlinear inflection points, saturation intervals, and optimal thresholds, while simultaneously assessing response variability using the scatter distribution. The core of PDP is calculating the average effect of model predictions on a target feature, defined as:

Equation 4 $${\hat{f}}_{s}\left(x_{s}\right)=E_{x_{c}}\left[\hat{f}\left(x_{s},x_{c}\right)\right]$$

where ${\hat{f}}_{s}\left(x_{s}\right)$ is the PDP output (average prediction at *x*_*s*_), $E_{x_{c}}\left[\cdot \right]$ is the expectation over all features other than *x*_*s*_, and $\hat{f}\left(x_{s},x_{c}\right)$ is the model’s prediction function.

For analyzing interaction mechanisms, we further calculated and interpreted SHAP Interaction Values to quantify coupling effects among morphological elements.^83^ We ranked global SHAP interaction values to identify the most influential feature combinations, thereby determining dominant synergistic or antagonistic patterns. Subsequently, interaction effect dependency plots were used for in-depth analysis. By examining the relationship between primary variables and interaction SHAP values, combined with secondary variables rendered via color, the specific pathways of synergistic emission amplification or antagonistic buffering are intuitively revealed.

#### Functional scenario evaluation

While the global model reveals the average impacts of UM on CE, these underlying mechanisms are likely modulated by urban function. Traditional grouped-modeling strategies often suffer from instability and bias due to sample imbalances. To overcome this, we employed a unified analytical framework where functional types are converted into one-hot encoded features and included alongside morphological indicators within a single XGBoost model.^84^ This strategy enables the model to autonomously learn complex interactions between morphological factors and functional attributes. Consequently, the functional-area-specific SHAP analysis presented here serves as a conditional interpretation of this unified model. By filtering sample subsets by function and examining SHAP value distributions, we rigorously dissected the model’s logic to reveal how the same morphological factor exerts differing impacts across distinct functional contexts.^85^

### Quantification and statistical analysis

We conducted data analysis using ArcGIS and Python. ArcGIS was used to preprocess spatial data and extract indicators. All raster datasets—land use, digital elevation model (DEM), nighttime light, and carbon emission grids—were uniformly projected and resampled. The Zonal Statistics as Table tool aggregated raster values to each block, calculating mean, sum, or majority for CE intensity, elevation, slope, aspect, GAR, WAR, and ANL. Building morphology indicators (e.g., BCD, BAD, FD, MND, BSC, SCD, BHI, SDBH, DCBH, FAR, SDBV, SVF) were computed from vector building data using geometry calculations and field statistics; MND was derived via the Near tool. Slope and aspect were generated from DEM using the Slope and Aspect tools. Spatial overlay analysis supported functional block identification, data management, and map production.

Python was used for statistical modeling, analysis, and visualization. We built an XGBoost regression model with the xgboost library to predict block-level CE intensity based on the multidimensional urban morphology indicators. Data preprocessing, including feature normalization and dataset splitting, was performed with scikit-learn. Hyperparameters were optimized using particle swarm optimization with 10-fold cross-validation on the training set. Model performance was evaluated on the test set using R^2^, RMSE, and MAE. To interpret the model, we applied the SHAP framework via the shap library, quantifying global feature importance, generating dependency plots to visualize nonlinear relationships and thresholds, and calculating interaction values to reveal synergistic and antagonistic effects. All statistical plots were produced with matplotlib and seaborn.
